# Different Neural Responses for Unfinished Sentence as a Conventional Indirect Refusal Between Native and Non-native Speakers: An Event-Related Potential Study

**DOI:** 10.3389/fpsyg.2022.806023

**Published:** 2022-03-03

**Authors:** Min Wang, Shingo Tokimoto, Ge Song, Takashi Ueno, Masatoshi Koizumi, Sachiko Kiyama

**Affiliations:** ^1^Department of Linguistics, Graduate School of Arts and Letters, Tohoku University, Sendai, Japan; ^2^Department of English Language Studies, Mejiro University, Tokyo, Japan; ^3^Department of Social Welfare, Faculty of Comprehensive Welfare, Tohoku Fukushi University, Sendai, Japan

**Keywords:** unfinished Japanese sentences, conventional indirect refusal, mentalization, P600, N400, P300, EPN, P200

## Abstract

Refusal is considered a face-threatening act (FTA), since it contradicts the inviter’s expectations. In the case of Japanese, native speakers (NS) are known to prefer to leave sentences unfinished for a conventional indirect refusal. Successful comprehension of this indirect refusal depends on whether the addressee is fully conventionalized to the preference for syntactic unfinishedness so that they can identify the true intention of the refusal. Then, non-native speakers (NNS) who are not fully accustomed to the convention may be confused by the indirect style. In the present study, we used event-related potentials (ERPs) of electroencephalography in an attempt to differentiate the neural substrates for perceiving unfinished sentences in a conventionalized indirect refusal as an FTA between NS and NNS, in terms of the unfinishedness and indirectness of the critical sentence. In addition, we examined the effects of individual differences in mentalization, or the theory of mind, which refers to the ability to infer the mental states of others. We found several different ERP effects for these refusals between NS and NNS. NNS induced stronger P600 effects for the unfinishedness of the refusal sentences, suggesting their perceived syntactic anomaly. This was not evoked in NS. NNS also revealed the effects of N400 and P300 for the indirectness of refusal sentences, which can be interpreted as their increased processing load for pragmatic processing in the inexperienced contextual flow. We further found that the NNS’s individual mentalizing ability correlates with the effect of N400 mentioned above, indicating that lower mentalizers evoke higher N400 for indirect refusal. NS, on the contrary, did not yield these effects reflecting the increased pragmatic processing load. Instead, they evoked earlier ERPs of early posterior negativity (EPN) and P200, both of which are known as indices of emotional processing, for finished sentences of refusal than for unfinished ones. We interpreted these effects as a NS’s dispreference for finished sentences to realize an FTA, given that unfinished sentences are considered more polite and more conventionalized in Japanese social encounters. Overall, these findings provide evidence that a syntactic anomaly inherent in a cultural convention as well as individual mentalizing ability plays an important role in understanding an indirect speech act of face-threatening refusal.

## Introduction

Every utterance has a possibility, to a greater or lesser degree, of threatening the addressee’s *face*, that is, their public self-image maintained in society (Brown and Levinson, 1987), and in turn can jeopardize the interpersonal relationship between the addresser and the addressee. In particular, the act of refusing an invitation is a face-threatening act (FTA) in that the refuser causes a serious conflict with the inviter’s friendly attitude. In order to redress the conflict, the refuser should use an appropriate politeness strategy to refuse indirectly, namely, by leaving the sentence unfinished to obscure their true intention of not performing the invited action. Although native speakers (NS) can easily understand polite strategies of indirect refusals, non-native speakers (NNS) often have trouble understanding such strategies as they are not fully accustomed to conventionally polite ways of hiding one’s true intention (Kasper, 1984).

The realization of indirect speech acts differs depending on the syntactic properties of each language. The salient characteristics of the Japanese language are: it has a head-final structure in which arguments of the subject (S) and the object (O) necessarily precede the head (i.e., verb: V); and in a compound sentence, a conjunction comes at the end of the preceding clause as shown in (1a), contrary to head-initial languages (e.g., English, Chinese) in which the conjunction comes at the beginning of a clause.


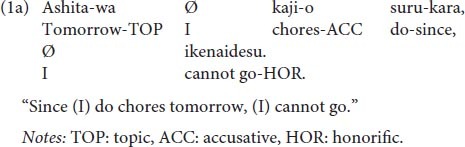


As in (1a), the preceding subordinate clause ending with the conjunction typically indicates the reason for refusal, while the subsequent main clause includes the true intention of the refusal itself. This syntactic property allows the Japanese language to easily omit the latter main clause including the negation of the invited action, such as *ikenaidesu* (meaning “I cannot go”) in (1b), resulting in an unfinished sentence with only a subordinate clause that ends with the conjunctive particle *-kara* (meaning “since”).


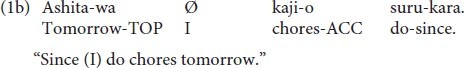


Although it is quite common in many languages to convey an FTA of refusal indirectly by only stating the reason not to do the invited action, as in (1b), it is characteristic of Japanese to leave the sentence unfinished by putting the conjunctive particle *-kara* (“since”) at the end of the sentence, although the particle syntactically requires the subsequent main clause. Japanese NS are known for their strong preference for this strategy of omitting the main clause, including their true intention, in order to indirectly realize FTAs in everyday conversations (Maynard, 1989). Notably, it is assumed that such unfinished sentences are not ambiguous for NS by virtue of conventionalization (Taguchi, 2014). Uttering such unfinished sentences can then be interpreted as a universal politeness strategy of performing an indirect speech act in order to be conventionally indirect (Brown and Levinson, 1987). However, NNS who are not fully accustomed to the conventionalized politeness strategy may find syntactically unfinished sentences that lack the main clause stating the speaker’s true intention of the FTA to be anomalous.

Experimental pragmaticists have demonstrated the complex linguistic and cognitive processes involved in the real-time comprehension of how conventionalized indirect speech acts are realized. Studies using electroencephalograms (EEG) indicate that NS recognize the intention of a speech act at a very early stage (Egorova et al., 2013; Gisladottir et al., 2015). Evidence from functional magnetic resonance imaging (fMRI) studies suggests that making inferences about indirect speech acts requires greater cognitive load, since such processing induces higher activation of regions of the brain, including the bilateral prefrontal cortex (Shibata et al., 2011; van Ackeren et al., 2012), the temporoparietal junction, and the medial prefrontal cortex (e.g., Bašnáková et al., 2013; Schurz et al., 2013; Feng et al., 2017), which are related to mentalization, cognitive empathy, or theory of mind (ToM), all of which are interchangeably used to refer to the capacity to understand other people by attributing mental states to them (Baron-Cohen, 1988). Although neurocognitive evidence for NNS’ processing of indirect speech acts is scarce, several behavioral studies suggest that it is difficult for NNS to comprehend indirect speech acts. Reaction time data showed that English NNS were not as fast or accurate in processing indirect requests and refusals as were NS (Taguchi, 2005). Holtgraves (2007) also indicated that NNS’ speech act recognition was not as reflexive as that of NS. However, these previous studies exclusively focused on European head-initial languages with SVO word orders. The influence of the syntactic structure of non-European head-final languages with SOV orders on the comprehension of indirect speech acts in NS and NNS remains unknown.

Utilizing high temporal resolution, event-related potentials (ERPs) of EEG provides useful findings for understanding the time course of online cognitive processing for the syntactic and pragmatic realizations mentioned above. From a syntactic viewpoint, the well-known ERP component of P600, which positively peaks approximately 600 ms after the given event, has been observed for the syntactic processing load across many languages, not just by NS but also by NNS (e.g., Frenck-Mestre et al., 2009; Gillon Dowens et al., 2010; Lemhöfer et al., 2014; Meulman et al., 2014). Regarding Japanese language processing, increasing numbers of ERP studies demonstrate higher cognitive load for complex syntactic processing in terms of word order (e.g., Yano et al., 2019) and syntactic island constraints (i.e., movement constraints on syntactic objects; Tokimoto et al., 2019). Furthermore, the functional role of the P600 has recently been required rethinking, such that it reflects integration difficulty in linguistic contexts rather than the interpretation of syntactic structure (e.g., Brouwer et al., 2012; Xiang et al., 2016), but these studies did not include Japanese. In order to differentiate the neural basis of pragmatic processing such as politeness judgments and that of syntactic processing, we need to consider the interplay of multiple ERP components, which depends on the cognitive demand of pragmatic and syntactic processing.

A variety of pragmatic realizations is known to induce another popular ERP component of negativity, N400, peaking at around 400 ms after stimulus onset. Although the N400 has traditionally been assumed to be an index of various kinds of semantic/pragmatic integration, such as inferring non-literal meanings (Pynte et al., 1996), retrieving conceptual information from context (see Canal and Bambini (2021) for a review), and irony comprehension (Katz et al., 2004), a recent parsimonious hypothesis of single-stream account for language processing suggests that the N400 reflects the retrieval of lexical information from memory, while the P600 relates to semantic/pragmatic processing instead (Brouwer et al., 2012). We should also notice that a robust N400 effect can be observed not only with visual stimuli, but also with auditory stimuli. This is relevant to the present study, which focuses on neural substrates for the pragmatic processing of indirect meanings of FTAs conveyed in spoken conversations. For example, a recent study reported an auditory N400 effect for processing non-literal meanings (Kessler et al., 2021). Another recent investigation compared the pragmatic N400 effect between English NS and the proficient NNS (Jankowiak et al., 2021), showing similarities between the two groups when comprehending figurative meanings. Taken together, these results indicate that the N400 effect should serve as a valid index reflecting both NS and NNS’ cognitive load when inferring indirect meanings from unfinished sentences of refusal within the auditory modality.

We may assume that if one speaks an unfinished sentence to indirectly realize an FTA in order to be polite to the addressee, such an unfinished sentence should mitigate the negative emotion postulated to be aroused in the addressee by virtue of FTA which could cause conflicts between the speaker and the addressee (e.g., Chen, 2013). In this context, NS are expected to promptly perceive such a politeness strategy, which might be reflected in an earlier neural response. Indeed, several neurolinguistic experiments of NS show that emotional processing via verbal information elicits earlier ERP components, such as early posterior negativity (EPN). Several studies have reported the EPN effect on emotional language processing (Schacht and Sommer, 2009; Mittermeier et al., 2011; Kissler and Herbert, 2013). With regards to Japanese, Kiyama et al. (2018) demonstrated the emotional effect of EPN on mood-modulating sentence-final particles. They found an individual difference in the EPN in terms of mentalization or ToM, indicating that Japanese NS with lower mentalizing ability yielded a greater EPN for complex processing of linguistic sentence-final particles that implicate the speaker’s emotions. The enhanced EPN in people with lower mentalizing ability may reflect their hypersensitivity to complex emotional linguistic markers which require flexible interpretation of the speaker’s attitudes. This suggests that low mentalizers are less able to grasp subtle indications of emotions, due to their stereotypical knowledge of these markers. Nevertheless, there is still a lack of neurolinguistic comparisons between NS and NNS concerning individual differences in emotional reactions to language.

Individual differences in mentalization during the processing of unfinished sentences of indirect refusals can also be expected because such sentences require the addressee’s ability to infer the speaker’s motivation and emotion for omitting the main clause indicating the intention of refusal. Several studies have indicated that participants with lower mentalizing ability took greater cognitive load for comprehending indirect requests (Trott and Bergen, 2019) and linguistic expressions in non-literal context (McKenna et al., 2015). This finding is in accordance with the increasing number of clinical pragmatic studies investigating syntactic and pragmatic (i.e., non-literal language processing) atypicality in adults and children with autism spectrum disorder (ASD), a diagnosis based on persistent deficits in mentalization or ToM and by restricted, repetitive patterns of behavior and interests (for a review see Cummings, 2014). Linguistic atypicality in people with ASD in Asian cultures is particularly evident in the processing of bound morphemes like sentence-final particles that have no substantial meaning but indicate the speaker’s subtle moods or emotions (e.g., Kamio, 1994; Matthews and Yip, 1994; Koo and Rhee, 2013).

In sum, this study investigates the neural substrates for perceiving sentences left unfinished to realize a conventionalized indirect speech act of refusal in Japanese, a head-final language. It focuses in particular on the effects of individual differences in mentalization. Specifically, we examine the following four hypotheses: The first hypothesis (H1), which focuses on syntactic unfinishedness, assumes that the P600 effect would be elicited by unfinished sentences that lack the main clause indicating the intention of refusal only in NNS. NS would not show a syntactic ERP effect because they automatically appreciate this highly conventionalized form of syntactic incompleteness, as it is fully conventionalized. The second hypothesis (H2) concerns the pragmatic processing of indirect refusal. We predicted that the N400 effect would be greater in NNS than in NS for unfinished sentences of indirect refusals as an FTA relative to finished sentences of direct refusals, since NS sub-unconsciously process such conventionalized indirect refusals. As for the third hypothesis (H3), which concerns the emotional reaction to the language form, we assumed that, compared to NNS from different cultural backgrounds, NS familiar with Japanese conventions would elicit earlier ERP components for emotional processing, such as EPN for finished sentences of direct refusals than for unfinished ones of indirect refusals. Lastly, with regards to our fourth hypothesis (H4), we expected that people with lower mentalizing ability would evoke greater amplitudes of the ERP components mentioned in the first three hypotheses, given lower mentalizers’ hypersensitivity to emotional language processing, as suggested in a previous study (Kiyama et al., 2018). The first three hypotheses entail a group comparison between NS and NNS, while the fourth requires a *post hoc* analysis to examine the effect of individual differences on the findings of the group comparisons. Conversely, these hypotheses concerning differences between NS and NNS would not be supported if the NNS have, not just advanced linguistic abilities such as precise syntax and a large vocabulary, but also a great aptitude to quickly learn the implicit pragmatic conventions of the Japanese language.

## Materials and Methods

### Participants

This study involved 30 Japanese NS and an equal number of NNS graduate and undergraduate students. Four participants were excluded from the data analysis owing to excessive EEG artifacts (*n* = 2) or participants’ inattention (*n* = 2), resulting in a final sample size of 28 participants in the NS group (14 men, mean age = 22.1 ± 2.3 years) and the NNS group (15 men, mean age = 24.8 ± 1.7 years). The NNS participants were highly proficient in Japanese because they used it in their daily communication. They had cleared the most difficult level of the Japanese-Language Proficiency Test, and they obtained a mean score of 74.0 (range: 60–88, *SD* = 7.3) out of 90 in the Tsukuba Test-Battery of Japanese (TTBJ). On average, they had studied Japanese for 5.8 years (*SD* = 2.8) and had stayed in Japan for at least 2.8 years (*SD* = 1.3) at the time of the experiment. The NNS participants were native Chinese speakers. All the participants (both the NS and NNS) were right-handed according to the FLANDERS handedness questionnaire (Nicholls et al., 2013; Okubo et al., 2014), and reported no neurological or psychiatric disorders. To measure individual mentalizing ability, we asked the participants to complete the standardized version of the Autism-Spectrum Quotient (AQ; Wakabayashi et al., 2006 for Japanese; Chan and Liu, 2008 for Chinese), which is known to substantially reflect the degree of mentalizing (ToM) ability within the general adult population (Baron-Cohen et al., 1997; Nieuwland et al., 2010). The average AQ (higher scores implicate a lower mentalizing ability) was 22.1 (*SD* = 7.1) of 50 for NS participants and 20.9 (*SD* = 6.0) for NNS participants. As shown in Table 1, these two groups did not have significant differences in AQ, sex ratio, handedness, and fluid intelligence, as revealed by the Raven’s Standard Progressive Matrices test (RSPM; Raven et al., 2003). All the participants provided written informed consent before the experiment which was conducted in accordance with the declaration of Helsinki, and received monetary compensation for their participation. The study was approved by the Ethics Committee of the Graduate School of Arts and Letters, Tohoku University, Japan.

**TABLE 1:** The properties of NS and NNS Japanese participants.

	NS (*n* = 28)	NNS (*n* = 28)	χ^2^/*t*	*df*	*p*
Sex ratio (men/women)	14/14	15/13	0.07	1	0.789
Mean age	22.2 (2.3)	24.8 (1.7)	5.18	50	0.000
Mean FLANDERS: handedness (Max = 10)	9.7 (0.6)	9.5 (1.0)	1.11	47	0.275
Mean AQ: reflecting mentalizing ability (Max = 50)	22.1 (7.1)	20.9 (6.0)	0.80	52	0.429
Mean RSPM: fluid intelligence (Max = 60)	57.1 (2.0)	57.0 (2.5)	0.17	52	0.864
Mean TTBJ: grammar of Japanese (Max = 90, NNS only)	–	74.0 (7.3)	–	–	–

*NS, native speakers; NNS, non-native speakers; AQ, Autism-Spectrum Quotient; RSPM, Raven’s Standard Progressive Matrices; TTBJ, Tsukuba Test Battery of Japanese.*

### Stimuli

We prepared 30 sets of stimulus dialogs in Japanese, each consisting of two turns of making an invitation and a refusal to elicit neural responses to the unfinished sentence indirectly expressing the speaker’s intention. In terms of whether the sentence has been finished or not, and whether or not the refusal is indirect, we manipulated sentence-final expressions of the second turn of refusal into three conditions: unfinished sentence of indirect refusal (USi) as a target condition, finished sentence of indirect refusal (FSi) as a syntactic control condition, and finished sentence of direct refusal (FSd) as a pragmatic (indirectness) control condition (Table 2). The USi has a clause indicating the reason for refusal (e.g., *Sonohi-wa ishogashii* meaning “I’m busy that day.”), ending with a conjunctive particle *-kara* (meaning “since”), as shown in (a) in Table 2. This is syntactically incomplete in that it only has a subordinate clause that ends with a conjunction, lacking the main clause. FSi as a syntactic control is identical to the USi as in Table 2 (b), except that the conjunction *-kara* was replaced with a sentence-final copula *-nda*, which does not require another clause. FSd, set as a pragmatic control condition as in Table 2 (c), is a complex sentence in which a subordinate clause identical with the USi precedes the main clause to directly indicate the intention of refusal (e.g., *Sonohi-wa ishogashii-kara, muri* meaning “Since I’m busy that day, it’s impossible”), as this expression is typically categorized as a direct refusal according to the semantic formula (Beebe et al., 1990) which is often utilized in cross-cultural pragmatic studies. Specifically, we expected that the comparison of USi with the syntactic control (FSi) would induce syntactic and emotional ERPs such as P600 and EPN (i.e., H1 and H3), while the comparison with the pragmatic control (FSd) concerns semantic/pragmatic ERPs like N400 (i.e., H2). We also prepared another 30 sets of filler dialogs, each consisting of a request and an acceptance or reservation. All stimulus dialogs and the English translation are available in Supplementary Material.

**TABLE 2:** Examples of stimulus dialogs in Japanese and the English translation.

First turn (Invitation)	Second turn (Refusal)	Condition
Nami, eiga-no chiketto-ga 2-mai aru-n-da-kedo, Nami, movie-GEN ticket-NOM 2-pieces be-NMLZ-COP-CONJ doyoobi issyoni mi-ni ika-nai? Saturday together see-DAT go-NEG “Nami (person name), (I) have two movie tickets. How about going for a movie together on Saturday?”	Aa, sonohi-wa isogasii-**kara**. Ah that day-TOP busy-since “Ah, since (I’m) busy that day.”	(a) USi (target)
	Aa, sonohi-wa isogasii-**n-da**. Ah that day-TOP busy-NMLZ-COP “Ah, (I’m) busy that day.”	(b) FSi (syntactic control)
	Aa, sonohi-wa isogasii-**kara, muri.** Ah that day-TOP busy-since impossible “Ah, since (I’m) busy that day, it’s impossible.”	(c) FSd (pragmatic control)

*Sentence-final expressions in bold were the critical expressions. USi, unfinished sentence of indirect refusal; FSi, finished sentence of indirect refusal; FSd, finished sentence of direct refusal; GEN, genitive; NOM, nominative; NMLZ, nominalizer; COP, copula; CONJ, conjunctive particle; DAT, dative; NEG, negative; TOP, topicalizer.*

These dialogs were selected based on a pilot test using auditory candidate materials of 36 target (i.e., invitation-refusal dialogs) sets as well as 36 filler sets, which were recorded by Japanese NS of the same gender (i.e., female-female or male-male) in their twenties. The speakers played a fixed role in the dialogs as either the inviter (requester) or the invitee (requestee) as naturally as possible. Their voices during role-playing were recorded using SFS/WASP version 1.54 (Huckvale, 2015) in a soundproof room. In the pilot test, 29 Japanese NS (14 men, mean age = 20.9 ± 1.3 years), who did not participate in the subsequent EEG experiment, were auditorily presented all the recorded stimuli, which were assessed in terms of naturalness and indirectness per dialog on a 6-point Likert scale (1: very unnatural/direct; 6: very natural/indirect). We excluded dialogs that indicated less than 5 on average from the naturalness scale. For the scale of indirectness, a one-way analysis of variance (ANOVA) [*F*_(1, 33)_ = 157.26, *p* < 0.001] and the multiple comparisons confirmed a significant difference between the indirect and direct conditions (i.e., FSd was assessed to be direct than USi; *p* < 0.001), whereas no significant difference was found between indirect conditions of USi and FSi (*p* = 0.570). To ensure that there was no confounding by prosody of the stimuli, we verified the differences in acoustic properties of the second sentences of the dialogs across the three conditions, following Paulmann et al. (2013). The results of one-way ANOVAs showed no significant differences (all *p*’s ns) across the conditions in terms of duration, highest and lowest pitch, or intensity, both in the critical part of the sentence-final expression and in the preceding part of the second sentence (Table 3).

**TABLE 3:** Acoustic characteristics of the second turn of the stimulus dialogs.

	The preceding part (e.g., *Aa, sonohi-wa isogasii* meaning “I’m busy that day”)					The critical sentence-final expression (the conjunction -*kara* or the copula -*nda*)				
	USi	FSi	FSd	*F*	*p*	USi (-*kara*)	FSi (-*nda*)	FSd (-*kara*)	*F*	*p*
	*M* (*SD*)	*M* (*SD*)	*M* (*SD*)			*M* (*SD*)	*M* (*SD*)	*M* (*SD*)		
Duration (ms)	2,344 (567)	2,375 (577)	2,418 (510)	0.11	0.860	323 (45)	322 (49)	320 (56)	0.41	0.591
Highest pitch (Hz)	280 (91)	298 (99)	271 (65)	0.62	0.716	240 (92)	200 (66)	213 (71)	1.67	0.623
Lowest pitch (Hz)	148 (53)	141 (54)	144 (53)	0.10	0.793	135 (54)	154 (57)	140 (44)	0.81	0.725
Intensity (dB)	76 (2)	77 (2)	76 (2)	0.86	0.480	76 (2)	77 (3)	76 (3)	0.55	0.329

*USi, unfinished sentence of indirect refusal; FSi, finished sentence of indirect refusal; FSd, finished sentence of direct refusal.*

### Procedure

**FIGURE 1:**
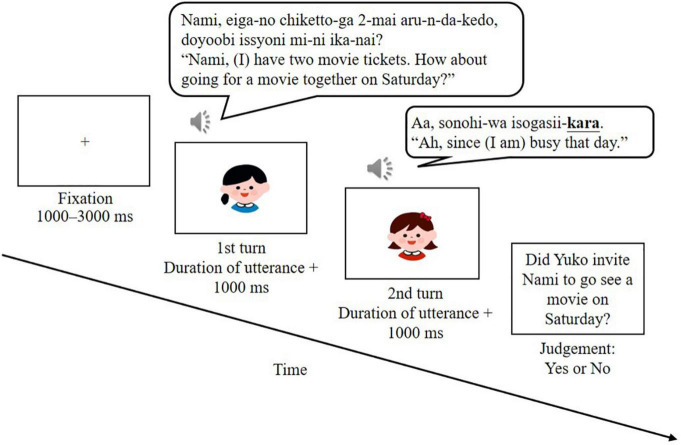
Experimental procedure for one trial. All events depicted were presented in Japanese in the actual experiment. A sentence-final expression *-kara* (“since”) or *-nda* (copula) at the 2nd turn of refusal was set as the critical expression of the ERP analysis.

The participants were asked to be seated in front of a computer screen (RDT196LM2, MITSUBISHI, Japan) in a dimly lit soundproofed room, listen to the dialog stimuli, and complete a content comprehension task of the dialogs, while their EEG were being recorded. As shown in Figure 1, each trial began with a fixation cross for the jittered time between 1,000 and 3,000 ms, followed by an auditory presentation of a two-turn dialog along with a visual presentation of pictures depicting the hypothetical characters. Each picture for each turn was presented for 1,000 ms more than the duration of each turn itself to create the natural flow of a conversation. The stimulus dialogs were auditorily presented through loudspeakers (Companion2 III BK, BOSE, United States). The volume of the acoustic stimuli was adjusted according to individual needs. To ensure that the participants were paying adequate attention to the dialogs, yes/no content comprehension questions were visually presented after one-third of all dialogs (both for target and filler ones). All trials were distributed within three blocks, with a small break between each block. The stimuli were presented in random order within each block. Each participant listened to one of the three types of the second turn set in a block. Before the commencement of the experiment, six practice trials were conducted. Stimulus presentation and behavioral data were obtained using E-Prime 3.0 (Psychology Software Tools, Pittsburgh, PA). The entire procedure for each participant lasted approximately 45 min on average. Immediately after the EEG measurement, the participants (both NS and NNS) were asked to assess the indirectness of one of the given stimulus dialogs for each condition on a 6-point Likert scale, as in the pilot test, to check our experimental manipulation.

### Electrophysiological Data Acquisition and Preprocessing

The EEG data were recorded from the Quick Amp EEG System (Brain Products, Munich, Germany) using 29 Ag/AgCl electrodes mounted on an elastic cap (EasyCap, Munich, Germany) according to the International 10/20 system. Two additional electrodes were attached to the upper orbital ridge and external canthi of the left eye to monitor the electrooculogram (EOG) and blink artifacts. The reference was set to the FCz online, and the EEG data were re-referenced offline to a common average reference. The impedances of most electrodes were maintained below 10 kΩ. Amplified analog voltages were digitized at 1,000 Hz with a system bandpass filter between 0 and 200 Hz. We set the triggers at the onset of the critical sentence-final expression (*-kara* or *-nda)* for the EEG recording.

Data preprocessing was realized offline using EEGLAB (Delorme and Makeig, 2004) implemented in Matlab R2015b (MathWorks, Natick, MA) in the following procedures. First, EEG data were downsampled to 250 Hz and high-pass filtered with a cutoff of 1 Hz. Subsequently, the power line noise was eliminated from the data using the CleanLine EEGLAB plug-in (Mullen, 2012). Artifact Subspace Reconstruction was performed to eliminate high-amplitude artifacts (Mullen et al., 2015). A channel was rejected if its correlation (*r*) with the surrounding channels was less than 0.8. Next, bad channels were interpolated using EEGLAB’s default spherical spline interpolation, and the data were re-referenced to a common average reference. An Adaptive Mixture Independent Component Analysis (Palmer et al., 2007) was performed for continuous EEG data to eliminate the remaining periodical artifacts. Segmentation was selected from –700 to 1,600 ms around the triggers. The data were further cleaned using a semi-automatic selection of independent components for artifact correction (Chaumon et al., 2015). Finally, epochs in which the EOG with amplitudes exceeded ± 70 μV were rejected. Individual channel artifacts led to the rejection of 2.9% of the data in NS and 4.3% in NNS.

### Statistical Analysis

Among the four hypotheses, the first three required group comparisons between NS and NNS to examine the ERP effects for the increased syntactic (H1), pragmatic (H2) and emotional (H3) processing load caused by the unfinishedness or indirectness of the sentence. H1 and H3 for the unfinishedness of syntactic structure were investigated by the comparison between USi and FSi, and H2 was examined via the USi and FSd comparison. Concerning H1, we investigated the P600 effect as an index for syntactic processing load, following its typical time window of 500–700 ms reported in a large number of previous studies (Kaan et al., 2000; Silva et al., 2017). To investigate H2, the pragmatic N400 effect was sought by focusing on its typical time window of 300–550 ms (Grillon et al., 1991; Baptista et al., 2018). As for H3, we explored earlier emotional ERP component of EPN, for which the typical time window was set at 150–400 ms (Mittermeier et al., 2011; Kiyama et al., 2018). Within each of these time windows, 2 (Unfinishedness: USi vs. FSi or Indirectness: USi vs. FSd) × 2 (NS vs. NNS) ANOVAs were conducted over the mean amplitudes of each of the 29 electrodes on the EEGLAB STUDY command structure (Delorme et al., 2011), with the former factor as a within-participant variable and the latter as a between-participant variable. When conducting this, non-parametric permutation (*n* = 2,000) statistics (Blair and Karniski, 1993) were computed with a false discovery rate (FDR) correction for multiple comparisons (Holm, 1979). Alpha level was set to 0.05. The baseline correction was done with the mean activity in the 100 ms after the end of the critical expression (i.e., *-kara* meaning “since” in USi and in FSd, *-nda* as a copula in FSi). We performed the post-stimulus baseline correction, instead of the pre-stimulus correction, following Tokimoto and Tokimoto (2018), given that the duration of the critical expressions across the three conditions of our stimuli was not completely the same as they were recorded separately.

Subsequently, we exported each participant’s information of the mean amplitude among the significant electrodes per condition from the EEGLAB STUDY, so that we conducted *post hoc* analyses to investigate the effects of individual mentalizing ability on the significant ERP effects for each of the above-mentioned time windows. For the *post hoc* analyses, we conducted analyses of covariance (ANCOVAs) for each participant’s mean amplitude of electrodes as the dependent variable, where two categorical independent variables of Unfinishedness/Indirectness and Group, a continuous independent variable (i.e., covariate) of AQ, and the two- and three-way interaction terms were included. The ANCOVAs were conducted on R version 4.1.1.

## Results

### Behavioral Data

Both the NS and NNS showed satisfactory performance in the content comprehension task, with a mean accuracy of 95.78% (*SD* = 0.20) for NS and 89.45% (*SD* = 0.31) for NNS. The content comprehension task was used to evaluate whether the participants had concentrated on the experiment. The overall high accuracy indicates that all participants were attentive to the task and adequately understood the dialogs, although the mean difference was significant between the two groups [*t*_(41)_ = 4.30, *p* < 0.001, *d* = 1.15].

### Electrophysiological Data

#### Comparison of Sentence Unfinishedness to Seek for Syntactic Processing: Unfinished Sentence of Indirect Refusal vs. Finished Sentence of Indirect Refusal

**FIGURE 2:**
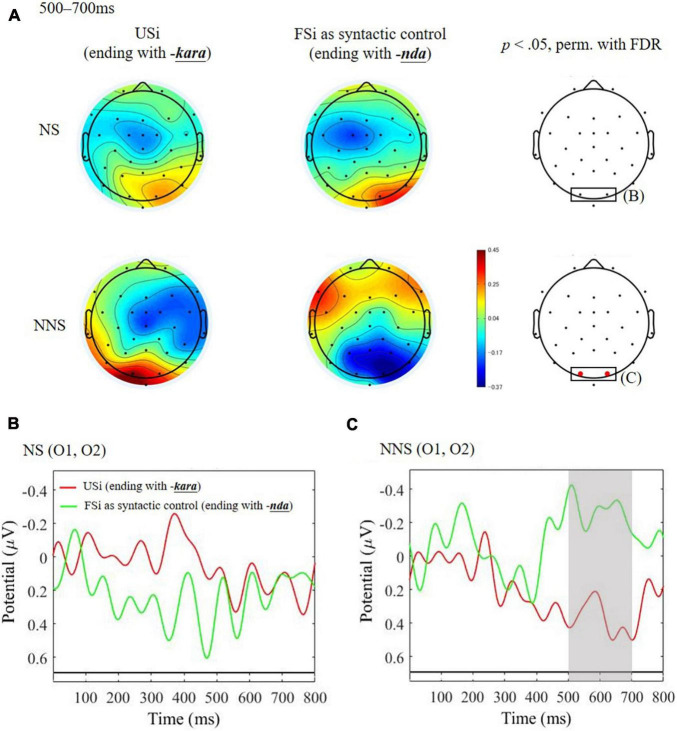
ERPs time-locked to the ending of the critical expression of unfinished Japanese sentence of indirect refusal (USi) and the finished sentence of indirect refusal (FSi) in native speakers (NS) and non-native speakers (NNS). **(A)** Mean topographies of ERP from 500 to 700 ms for USi and FSi in NS and NNS and the results of statistical tests (*p* < 0.05 of permutation test with false-discovery rate: FDR) are shown on the right side, with electrodes indicating significance (O1 and O2) in red. **(B)** Mean ERP amplitudes at the occipital electrodes (O1 and O2) in NS. **(C)** Mean ERP amplitudes at the occipital electrodes (O1 and O2) in NNS. The gray bar indicates the latency showing a significant difference between USi and FSi.

Figure 2 shows the result of the group comparison to seek for the syntactic ERP effect caused by the Unfinishedness of the sentence of indirect refusals. NNS showed a significantly distinct amplitude at occipital electrode sites (O1 and O2), with an enhanced positive amplitude induced by USi as compared to its syntactic control of FSi during the time window of 500–700 ms (Figures 2A,C). This finding is consistent with the P600 effect. However, the effect was not significant in NS (Figures 2A,B). A *post hoc* ANCOVA for the mean amplitude of the time-window (Table 4) to seek for the individual difference found no significant effect of individual AQ on the NNS’s P600 effect; this was based on the result of the three-way interaction of Unfinishedness by Group by AQ [*F*_(1, 104)_ = 0.90, *p* = 0.345, η^2^ = 0.001] as well as the two-way interaction of Unfinishedness by AQ [*F*_(1, 104)_ = 0.79, *p* = 0.375, η^2^ = 0.006].

**TABLE 4:** Results of analysis of covariance for ERP amplitude in the 500–700 ms time window between the unfinishedness comparison of USi and FSi.

Contrast	*df*	*MS*	*F*	*p*	η^2^
Unfinishedness (USi vs. FSi)	1	4.02	4.29	0.041	0.036
Group (NS vs. NNS)	1	1.20	1.17	0.282	0.010
AQ	1	0.56	0.60	0.440	0.005
Unfinishedness by Group	1	6.31	6.74	0.011	0.054
Unfinishedness by AQ	1	0.74	0.79	0.375	0.006
Group by AQ	1	0.09	0.09	0.763	0.001
Unfinishedness by Group by AQ	1	0.84	0.90	0.345	0.001
Error	104	0.94			

*USi, unfinished sentence of indirect refusal; FSi, finished sentence of indirect refusal; NS, native speakers; NNS, non-native speakers; AQ, Autism-Spectrum Quotient.*

#### Comparison of Sentence Indirectness to Seek for Pragmatic Processing: Unfinished Sentence of Indirect Refusal vs. Finished Sentence of Direct Refusal

**FIGURE 3:**
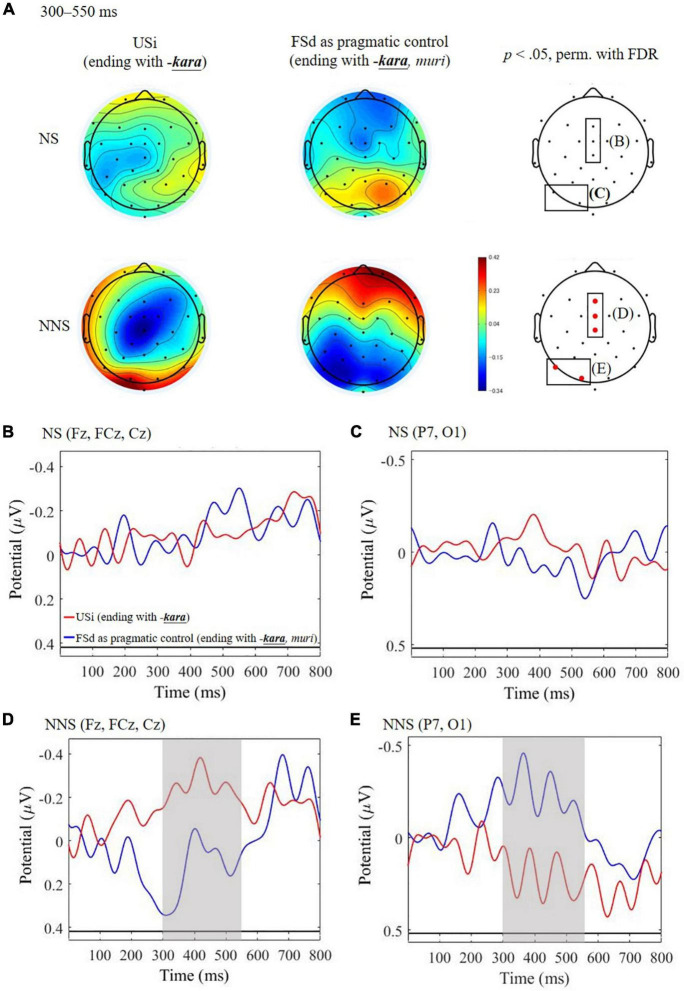
ERPs time-locked to the ending of the critical expression of unfinished Japanese sentence of indirect refusal (USi) and the finished sentence of direct refusal (FSd) in native speakers (NS) and non-native speakers (NNS). **(A)** Mean topographies of ERP from 300 to 550 ms for USi and FSd in NS and NNS and the results of statistical tests (*p* < 0.05 of permutation test with false-discovery rate: FDR) are shown on the right side, with electrodes indicating significance (Fz, FCz, Cz, P7, and O1) in red. **(B)** Mean ERP amplitudes at the central electrodes (Fz, FCz, and Cz) in NS. **(C)** Mean ERP amplitudes at the left occipital electrodes (P7 and O1) in NS. **(D)** Mean ERP amplitudes at the central electrodes (Fz, FCz, and Cz) in NNS. **(E)** Mean ERP amplitudes at the left occipital electrodes (P7 and O1) in NNS. The gray bars indicate the latencies showing significant differences between USi and FSd.

**FIGURE 4:**
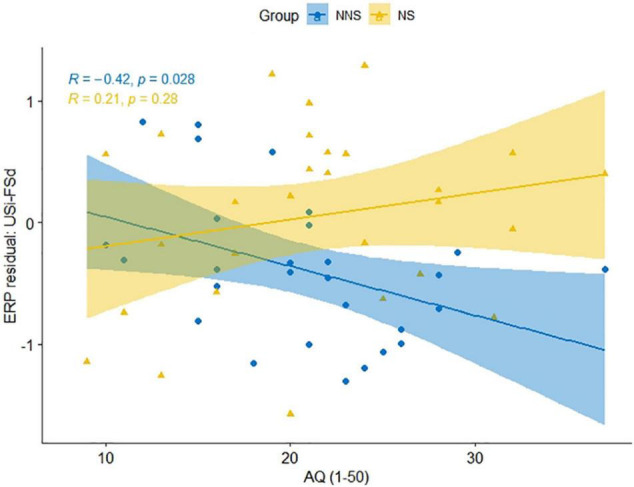
Plot of Autism-Spectrum Quotient (AQ) and the residuals from ERP amplitude for unfinished Japanese sentence of indirect refusal (USi) minus the finished sentence of direct refusal (FSd) within the 300–550 ms time window in native speakers (NS) and non-native speakers (NNS).

The result of the group comparison of the USi with the pragmatic control FSd in terms of the Indirectness of the refusal sentence is presented in Figure 3. We found that the NNS yielded an enhanced negative amplitude for USi than FSd in the central region (Fz, FCz, and Cz, Figures 3A,D), and also an increased positive amplitude in the left occipital region (P7 and O1, Figures 3A,E), during the time-window of 300–550 ms. The negative component is considered as the N400 effect, as we hypothesized, whereas the positivity was not expected. In NS, on the other hand, the both negative and positive deflections were not significant (Figures 3A–C). Further, as a result of a *post hoc* ANCOVA (Table 5), we found a significant three-way interaction [*F*_(1, 104)_ = 4.87, *p* = 0.023, η^2^ = 0.044] among Indirectness, Group, and AQ, indicating that NNS with lower AQ (reflecting higher mentalizing ability) yielded greater N400 for the USi in comparison with FSd (*r* = –0.42, *p* = 0.028), whereas NS did not indicate significant correlation (*r* = 0.21, *p* = 0.280), as plotted in Figure 4.

**TABLE 5:** Results of analysis of covariance for ERP amplitude in the 300–550 ms time window for the indirectness comparison of USi and FSd.

Contrast	*df*	*MS*	*F*	*p*	η^2^
Indirectness (USi vs. FSd)	1	3.35	3.69	0.058	0.030
Group (NS vs. NNS)	1	0.12	0.13	0.714	0.001
AQ	1	0.51	0.57	0.453	0.004
Indirectness by Group	1	5.78	6.37	0.013	0.053
Indirectness by AQ	1	0.10	0.11	0.745	0.001
Group by AQ	1	1.76	1.94	0.167	0.016
Indirectness by Group by AQ	1	4.87	5.36	0.023	0.044
Error	104	0.91			

*USi, unfinished sentence of indirect refusal; FSd, finished sentence of direct refusal; NS, native speakers; NNS, non-native speakers; AQ, Autism-Spectrum Quotient.*

#### Comparison of Sentence Unfinishedness to Seek for the Emotional Processing: Unfinished Sentence of Indirect Refusal vs. Finished Sentence of Indirect Refusal

**FIGURE 5:**
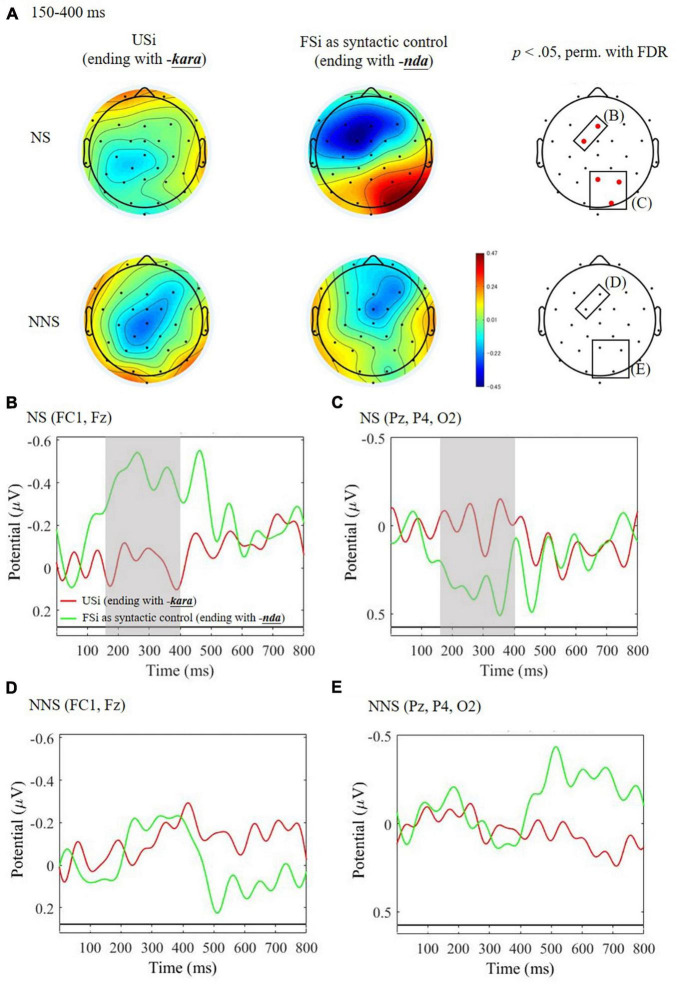
ERPs time-locked to the ending of the critical word of unfinished Japanese sentence of indirect refusal (USi) and the finished sentence of indirect refusal (FSi) in native speakers (NS) and non-native speakers (NNS). **(A)** Mean topographies of ERP from 150 to 400 ms for USi and FSi in NS and NNS and the results of statistical tests (*p* < 0.05 of permutation test with false-discovery rate: FDR) on the right side, with electrodes indicating significance (FC1, Fz, Pz, P4, and O2) in red. **(B)** Mean ERP amplitudes at the parietal electrodes (Pz, P4, and O2) in NS. **(C)** Mean ERP amplitudes at the frontal electrodes (FC1 and Fz) in NS. **(D)** Mean ERP amplitudes at the parietal electrodes (Pz, P4, and O2) in NNS. **(E)** Mean ERP amplitudes at the frontal electrodes (FC1 and Fz) in NNS. The gray bars indicate the latencies showing significant differences between USi and FSi.

Figure 5 shows the result of the group comparison of the USi with the FSi, in terms of Unfinishedness, to examine the enhanced emotional processing load in the earlier time window of 150–400 ms after the critical expression. In this time window, NS yielded a greater negative amplitude for FSi than USi in the frontal region (FC1 and Fz, Figures 5A,B), as well as a greater positive amplitude in the parietal and occipital regions (Pz, P4, and O2, Figures 5A,C). This negativity is in accordance with the predicted EPN effect, while the positivity was not as we hypothesized. NNS, contrarily, did not reveal any significant early effects (Figures 5A,D,E). The *post hoc* ANCOVA to seek for the individual AQ difference (Table 6) revealed no significant two- and three-way interaction effects concerning AQ [*F*_(1, 104)_ = 1.30, *p* = 0.257, η^2^ = 0.011 for Unfinishedness by AQ; *F*_(1, 104)_ = 0.01, *p* = 0.987, η^2^ < 0.001 for Unfinishedness by Group by AQ].

**TABLE 6:** Results of analysis of covariance for ERP amplitude in the 150–400 ms time window between the unfinishedness comparison of USi and FSi.

Contrast	*df*	*MS*	*F*	*p*	η^2^
Unfinishedness (USi vs. FSi)	1	3.76	4.17	0.040	0.034
Group (NS vs. NNS)	1	3.03	3.36	0.069	0.027
AQ	1	0.24	0.27	0.605	0.002
Unfinishedness by group	1	3.55	3.93	0.050	0.034
Unfinishedness by AQ	1	1.17	1.30	0.257	0.011
Group by AQ	1	5.43	6.02	0.016	0.058
Unfinishedness by Group by AQ	1	0.01	0.01	0.978	<0.001
Error	104	0.90			

*USi, unfinished sentence of indirect refusal; FSi, finished sentence of indirect refusal; NS, native speakers; NNS, non-native speakers; AQ, Autism-Spectrum Quotient.*

### Manipulation Check of Indirectness of the Stimuli

The NS and NNS participants’ rating for the Indirectness of the stimulus sentences of refusal is generally consistent with that of the pilot study completed by another NS. A 3 (USi, FSi, and FSd) × 2 (NS and NNS) ANOVA for the assessed indirectness (z-score obtained from a 6-point Likert scale from 1: direct to 6: indirect) revealed that both NS and NNS similarly differentiated the degree of indirectness across the three conditions, as the main effect of stimulus type was significant [*F*_(2, 162)_ = 80.97, *p* < 0.001, η^2^ = 0.428], while the main effect of group [*F*_(1, 54)_ = 2.944, *p* = 0.091, η^2^ = 0.015] and their interaction [*F*_(2, 162)_ = 0.01, *p* = 0.995, η^2^ < 0.001] were not significant. The multiple comparison corrected with Bonferroni method indicated that both NS and NNS assessed FSd to be significantly more direct (*M* = –0.91 for NS, *M* = –0.93 for NNS; *p* < 0.001), in comparison with other two indirect conditions of USi (*M* = 0.49 for NS, *M* = 0.50 for NNS) and FSi (*M* = 0.42 for NS, *M* = 0.43 for NNS). The difference between USi and FSi was not significant (*p* = 0.531).

## Discussion

The present ERP study examined four hypotheses to compare NS and NNS as well as the individual difference in mentalization for processing of indirect refusals conventionalized in Japanese. As a result, the neural substrates for perceiving unfinished sentences to perform a conventionalized indirect refusal as an FTA have been elucidated, even though the subjective assessment for the unfinished sentences is not significantly different between the two groups. H1 concerning syntactic unfinishedness, H2 for pragmatic processing of indirectness of the refusal, and H3 regarding emotional reaction to the unfinishedness were generally supported, while H4 was only partially supported.

First, as expected in H1, we observed the P600 effect for processing unfinished sentences compared to finished sentences only in NNS, whereas this effect was not found in NS. Although the P600 effect has been consistently reported for syntactic anomalies in Japanese (Tokimoto et al., 2019; Yano et al., 2019), the present study found the P600 to be prevalent for NNS’s processing of unfinished Japanese sentences in indirectly realizing a speech act of refusal. This suggests that NNS of Japanese particularly experience a processing difficulty with this kind of unfinished sentence. Conversely, it is obvious that the NS, who did not yield the P600, may process these sentences in a different manner. The finding that the NS may not consider the unfinished sentences anomalous is consistent with the previous theoretical assumption that Japanese NS are accustomed to syntactic unfinishedness in face-threatening situations, as they prefer to obscure the true (impolite) intentions. The difference in the P600 effect between NS and NNS supports NS of Japanese’s automatic processing of unfinished sentences ending with a conjunctive particle (*-kara* meaning “since”) in the situation of refusals.

Particularly, our NNS participants were native speakers of Chinese, which has a different syntactic property from Japanese. In Japanese, the structure of a subordinate clause ending with a conjunction is commonly used, while the structure of the Chinese subordinate clause requires a conjunction coming at the beginning of a clause (Yap et al., 2014). This difference reflects whether the language is consistently head-initial (like Chinese) or head-final (like Japanese) at all phrasal levels. It is known that head-final languages typically have case-marking information, which enables incremental structure building in a language with flexible word orders (Kazanina, 2017). Given a large number of psycholinguistic experiments (e.g., Kamide, 2008; Sato et al., 2013; Kurumada and Jaeger, 2015; Witzel and Witzel, 2016) demonstrating that NS of Japanese as a head-final language have an advanced syntactic ability of anticipatory sentence processing, Japanese NS may be predicting the contents of the latter main clause of a complex sentence much earlier, while they are presented with the preceding subordinate clause. Chinese, contrarily, is categorized as a head-initial language without case markers and flexible word order, which may influence the processing difficulty of Chinese learners of Japanese inferring the omitted main clause of complex sentences in Japanese.

Consistent with H2 with regard to pragmatic processing, the N400 effect was elicited for the indirect speech act of refusal compared to the direct speech act only in NNS. This finding supports the notion that NNS with different cultural conventions need more effort to understand indirect speech acts. Even if they are highly proficient in the target language, NNS should rely on explicit verbal cues to recognize the speaker’s true intentions. The present experimental design prepared the pragmatic control condition of FSd as a direct speech act of refusal by adding the main clause stating the direct refusal meaning (e.g., “It’s impossible”) after the preceding subordinate clause stating the reason to refuse (e.g., “I’m busy that day”), where the NNS participants could use the main clause to specify the speech act. However, the target condition of the USi lacked the main clause indicating the refusal intention. Presumably, the lack of the main clause causes NNS to experience difficulty inferring the illocutionary force of a refusal. NS, contrarily, did not show the enhanced load of processing of the indirect speech act of refusal, suggesting that they have no extra cognitive load for indirect speech act recognition. The finding may reflect that these unfinished Japanese sentences of indirect refusals are highly conventionalized in NS of Japanese.

Furthermore, within the same time window of 300–500 ms, another unexpected positive component was induced for the same comparison of the USi with the FSd as a pragmatic control. Although caution is advised, this positivity may be related to the P300, which is known to be evoked by the oddball paradigm (e.g., Polich and Margala, 1997; Katayama and Polich, 1999; Herrmann and Knight, 2001), which detects attention for improvable stimuli compared with provable ones. Especially in the linguistic vein, the oddball P300 is reported to reflect the mental representation for context updates (Donchin and Coles, 1986), in that newly given improvable linguistic contexts require updating the acquired mental representation for provable contexts. In light of this, the USi that evoked P300 compared to the FSd may result from an unexpected flow of dialog for NNS, presumably because they experience a kind of context mismatch in which they prefer a direct sentence with an explicit refusal, rather than an indirect sentence obscuring the true intention.

Concerning H3 for emotional processing for whether the indirect refusal is finished or unfinished, only NS elicited significant ERP effects of both positive and negative deflections for the FSi compared to the USi. However, NNS did not yield any earlier ERP effects. We interpret the NS’s significant negativity as EPN in accordance with H3, and the positivity as P200, another well-known early ERP index for emotional processing, because the P200 is reportedly dependent on the emotional congruency of words within the context (León et al., 2010; Leuthold et al., 2015), and because the effect is known to respond to auditory language stimuli as well as visual stimuli (Paulmann et al., 2008). These NS’s ERP effects for the FSi in earlier time window suggest their negative emotional reaction to letting sentences finished, given that NS of Japanese are especially reluctant to complete sentences themselves. For NS, such finished sentences may act as less polite and less conventionalized strategies for performing an indirect refusal. Rather, they clearly value unfinished sentences, as the politeness theory (Brown and Levinson, 1987) argued that uttering unfinished sentences functions as a kind of politeness strategy. NNS who are not fully accustomed to Japanese culture, on the other hand, may not be able to distinguish the politeness functions between finished and unfinished sentences. This NNS’s insensitivity to Japanese politeness might have resulted in no significant effects concerning emotional ERPs, but it yielded a syntactic anomaly (i.e., P600) for the forms at the sentence-final position.

Lastly, in terms of H4 for individual differences in mentalization, we found a correlation between AQ and the amplitude of N400 only in NNS’s processing for perceiving indirect refusals. Namely, NNS participants with higher AQ (reflecting lower mentalizing ability) evoked larger amplitudes of the N400 for unfinished sentences to convey the intention of indirect refusal, compared to finished sentences as direct refusals, whereas NS did not indicate such correlation. The result suggests that NNS with lower mentalizing abilities require more cognitive loads to infer the indirect intention of unfinished sentences of face-threatening refusals. NS, on the other hand, did not indicate individual differences in mentalization for any ERP effects for syntactic, pragmatic, and emotional processing. This was inconsistent with the previous ERP study (Kiyama et al., 2018), which found that NS’s individual mentalizing ability modulated the effects of the emotional component of EPN for processing Japanese sentence-final particles depending on context. The present study’s manipulation of sentence-final expressions may not be distinctive enough for NS of Japanese, because the preceding context was identical across the three conditions of USi, FSi, and FSd, unlike Kiyama et al. (2018).

Overall, the present study found opposing ERP effects for unfinished sentences of refusal between NS and NNS. Namely, NS seem to smoothly process the unfinished sentences and feel anomalous with finished ones, while NNS may experience a higher syntactic and semantic cognitive load for unfinished sentences. The unfinished sentences utilized in this experiment were syntactically incomplete, ending with a conjunction *-kara* (meaning “since”) without the following main clause. The finding that NS did not experience a syntactic anomaly despite this unfinishedness supports the supposition that some conjunctions in contemporary Japanese do not necessarily function as conjunctions, but are often perceived as a kind of mood-expressing sentence-final particle, as the conjunctions are placed at the end of the sentence (National Institute for Japanese Language and Linguistics, 1951; Haugh, 2008). This ongoing syntactic/semantic status change of conjunctions in Japanese are not limited to the particle *-kara*, but also other particles including *-node* (meaning “since”), *-shi* (meaning “and”), *-kedo* (meaning “but”), presumably reflecting Japanese NS’ reluctance to make strong assertions through the single speaker finishing a sentence (National Institute for Japanese Language and Linguistics, 1951; Haugh, 2008). It has long been assumed that Japanese NS prefer to leave sentences unfinished, expecting the addressee to complete them, so that the speaker and the addressee collaborate to make a sentence together. This phenomenon is known as co-construction (Mizutani, 1980). Indeed, many qualitative linguistic studies of conversation analysis observe the complex turn-taking patterns in Japanese conversations, including co-constructions and long, twisted sentences allowing the addressee’s overlapping inputs (Tanaka, 1999; Hayashi, 2003). Even though the style may be inherent in Japanese culture, it is unsurprising that NNS have particular difficulty following such chaotic structures of conversation. Here lies potential for misunderstanding between Japanese NS and NNS, such that NS may feel uncomfortable with NNS’s finished sentences and might unfairly perceive them as impolite. This study provided the first neurophysiological evidence concerning these potential misunderstandings resulting from Japanese preferences for unfinished sentences, serving as a reference especially for adult learners of Japanese who have already acquired their own pragmatic language skills inherent in their native cultures.

Nonetheless, the current study contains several limitations, which require further examination in future studies. First, as our initial goal was to differentiate the neural responses to the syntactic unfinishedness in conveying refusals as an FTA between NS and NNS, we focused on the effect of the syntactic properties of head-directionality of languages, namely, whether each group’s native language was head-initial or head-final. However, the preference for syntactic unfinishedness when realizing FTAs is heavily influenced by different cultural backgrounds, besides the syntactic properties *per se*. As indicated by the dichotomy between individualism and collectivism (Hall, 1976; Triandis and Gelfand, 1998), it is assumed that people from individualistic cultures emphasize self-consciousness, while those from collectivistic cultures focus on interdependence in interpersonal interactions. These differential patterns of behavior and thinking would determine whether those peoples prefer to leave sentences unfinished in order to perform indirect speech acts (Searle, 1969) to impress polite addressees. Although our participants were Japanese NS and NNS whose native language was Chinese, both within the alleged collectivistic cultures, future research should compare these effects with NNS from individualistic cultures. Of course, the realization of refusal in actual encounters depends on how the preceding invitation was conveyed. To better understand the neural substrates for processing refusals, we should investigate the neural responses to the speech acts such as invitation and request preceding the refusal, which are significantly influenced by the cultural characterization as well (e.g., Holtgraves and Joong-Nam, 1990 for request; Kiyama et al., 2019 for invitation). The next concern is about the NNS’s effect on individual differences regarding the attitude of mentalization among typically developed young adults utilizing AQ as a convenient screening questionnaire. To confirm the current finding about the correlation between individual AQ and the N400 effect reflecting the pragmatic anomaly for the unfinished indirect sentences of refusals, we need to expand studies of the neural basis for indirect speech acts to include people with ASD. Naturally, we should differentiate the interaction effect of autistic traits or mentalizing ability with the sex difference, in the light of an apparent male bias in ASD prevalence (e.g., Werling and Geschwind, 2013). Further, the present ERP experiment was limited to a relatively small number of participants (i.e., *n* = 28 for each group) and item number of stimulus dialogs (i.e., 30 sets). Particularly, the less sufficient number of participants could obscure the tendency of individual differences. Hence, we should exercise caution in our current interpretation of the ERP findings, particularly concerning its correlation with the individual difference in mentalization. Further replication studies will contribute to better understand more plausible ways of influences of individual differences in cognitive preferences on the comprehension of indirect speech acts.

## Conclusion

This electrophysiological study first demonstrated that the neural response to unfinished sentences to perform a conventionalized indirect speech act of refusal differs between NS and NNS of Japanese, which is representative of head-final languages. NNS, who are not accustomed to the syntactic property, appear to find the syntactic structure anomalous and experience an increased cognitive load when processing unfinished sentences, implying an indirect refusal in a conventionalized manner. Further, the NNS’s individual mentalizing ability seems to boost their sensitivity to the pragmatic anomaly of indirect refusal in an unfinished sentence, presumably because higher mentalizers have sensitive awareness of sentence-final expressions in addition to the proposition of a sentence. Conversely, NS of Japanese inherent in the cultural convention did not induce such pragmatic cognitive loads for unfinished sentences when performing a conventionalized indirect refusal. Overall, these findings provide evidence that a syntactic anomaly inherent in a cultural convention and individual mentalizing ability play an important role in comprehending an indirect speech act of face-threatening refusal.

## Data Availability Statement

The datasets generated during the current study are available from the corresponding author on reasonable request.

## Ethics Statement

The studies involving human participants were reviewed and approved by the Ethics Committee of the Graduate School of Arts and Letters, Tohoku University, Japan. The participants provided their written informed consent to participate in this study.

## Author Contributions

MW, ST, GS, MK, and SK contributed to the conception and design of the study. MW, GS, and SK collected the data. MW, ST, TU, and SK ran the data analysis. MW, ST, MK, and SK interpreted the data and discussed the results. MW and SK wrote the manuscript. ST, GS, TU, and MK review the manuscript. MK and SK acquired funding. SK supervised the project. All authors participated in interpreting the findings and approved the content of the manuscript.

## Conflict of Interest

The authors declare that the research was conducted in the absence of any commercial or financial relationships that could be construed as a potential conflict of interest.

## Publisher’s Note

All claims expressed in this article are solely those of the authors and do not necessarily represent those of their affiliated organizations, or those of the publisher, the editors and the reviewers. Any product that may be evaluated in this article, or claim that may be made by its manufacturer, is not guaranteed or endorsed by the publisher.
