# Supplementary material for: Different Neural Responses for Unfinished Sentence as a Conventional Indirect Refusal Between Native and Non-native Speakers: An Event-Related Potential Study
Source: Front Psychol. 2022 Mar 3;13:806023. doi: 10.3389/fpsyg.2022.806023 (PMC8929272; doi:10.3389/fpsyg.2022.806023)
Supplement: Supplementary file 1 [file Table_1.docx]

Supplementary data: Stimulus dialog sets used for the ERP experiment

Table 1. Stimulus dialog sets of invitation–refusal exchange used in the experiment. Each set consists of two turns of making an invitation and a refusal in terms of three conditions: unfinished sentence of indirect refusal (USi), finished sentence of indirect refusal (FSi), finished sentence of direct refusal (FSd).

| Item | First turn | Second turn | Condition |
| --- | --- | --- | --- |
| 1 | Nami, eiga-no chiketto-ga 2-mai aru-n-da-kedo, doyobi issyoni mi-ni ika-nai?  “Nami, (I) have two movie tickets, how about going for a movie together Saturday?” | Aa, sonohi-wa isogashii kara*.*  “Ah, since (I’m) busy that day.” | USi |
|  |  | Aa, sonohi-wa isogashii-n-da*.*  “Ah, (I’m) busy that day.” | FSi |
|  |  | Aa, sonohi-wa isogashii kara muri.  “Ah, it’s impossible since (I’m) busy that day.” | FSd |
| 2 | Yūko, raishū gyouza matsuri aru-n-da-kedo, issyoni ika-nai?  “Yūko, there's a dumpling festival next week, would you like to come together?” | Aa, jikka-ni kaera-nai-to i-ke-nai kara  “Ah, since (I) have to go back to my parents’ house.” | USi |
|  |  | Aa, jikka-ni kaera-nai-to i-ke-nai-n-da.  “Ah, (I) have to go back to my parents’ house.” | FSi |
|  |  | Aa, jikka-ni kaera-nai-to i-ke-nai kara muri.  “Ah, it’s impossible since (I) have to go back to my parents’ house.” | FSd |
| 3 | Nami, karaoke-ni iki-tai-n-da-kedo, syumatu issyoni ika-nai?  “Nami, (I) wanna go to karaoke, would you like to come together at the weekend?” | Aa, syumatu-ha youji-ga aru kara.  “Ah, since (I) have things to do at the weekend.” | USi |
|  |  | Aa, syumatu-ha youji-ga aru-n-da.  “Ah, (I) have things to do at the weekend.” | FSi |
|  |  | Aa, syumatu-ha youji-ga aru kara muri.  “Ah, it’s impossible since (I) have things to do at the weekend.” | FSd |
| 4 | Yūko, kondo-no nichiyōbi, isshoni tsuri-ni ika-nai?  “Yūko, how about going fishing together this Sunday?” | Aa, zemi-no happyou-no junbisuru kara.  “Ah, since (I) have to prepare a presentation for my seminar.” | USi |
|  |  | Aa, zemi-no happyou-no junbisuru-n-da.  “Ah, (I) have to prepare a presentation for my seminar.” | FSi |
|  |  | Aa, zemi-no happyou-no junbi surukara muri.  “Ah, it’s impossible since (I) have to prepare a presentation for my seminar.” | FSd |
| 5 | Nami, kotoshi-no hanabi daikai sugoi rashi-yo. raisyū issyoni mi-ni ika-nai?  “Nami, I heard that the fireworks display this year is amazing. How about going to see it together next week?” | Aa, oya-to issyoni ryukosuru kara.  “Ah, since (I) will travelling with my parents.” | USi |
|  |  | Aa, oya-to issyoni ryukosuru-n-da.  “Ah, (I) will travelling with my parents.” | FSi |
|  |  | Aa, oya-to issyoni ryukosuru kara muri.  “Ah, it’s impossible since (I) will travelling with my parents.” | FSd |
| 6 | Yūko, fuyuyasumi-ni nat-tara, issyoni onsen ika-nai?  “Yūko, would you like to go to the hot springs together over winter break?” | Aa, baito isougashii kara.  “Ah, since (I’m) busy with my part-time job.” | USi |
|  |  | Aa, baito isogashii-n-da.  “Ah, (I’m) busy with my part-time job.” | FSi |
|  |  | Aa, baito isogashii kara muri.  “Ah, it’s impossible since (I’m) busy with my part-time job.” | FSd |
| 7 | Nami, atarashi ramenya-ga deki-ta-n-da-yo. Raisyu issyoni tabe-ni ika-nai?  “Nami, there's a new noodle shop. would you like to go eating together next week?” | Aa, raisyu-ha jikken-ga isogashii kara.  “Ah, since (I'm) busy with experiments next week.” | USi |
|  |  | Aa, raisyu-ha jikken-ga isogashii-n-da.  “Ah, (I'm) busy with experiments next week.” | FSi |
|  |  | Aa, raisyu-ha jikken-ga isogashii kara muri.  “Ah, it’s impossible since (I'm) busy with experiments next week.” | FSd |
| 8 | Yūko, atsuku nat-teki-ta kara tisyatu kai-tai-n-da. Nichiyōbi issyoni ika-nai?  “Yūko, I want to buy a T-shirt since it' s getting hot. Would do you like to go together on Sunday?” | N, motachi-to karaoke-no yakusokusi-ta kara.  “Hmm, since (I) promised to go to karaoke with my friends.” | USi |
|  |  | N, omotachi-to karaoke-no yakusokusi-ta-n-da.  “Hmm, (I) promised to go to karaoke with my friends.” | FSi |
|  |  | N, omotachi-to karaoke-no yakusokusi-ta kara muri.  “Hmm, it’s impossible since (I) promised to go to karaoke with my friends.” | FSd |
| 9 | Nami, asobi-ni ikitai-naa. Doyōbi issyoni suizokukan ika-nai?  “Nami, I want to go hang out. Would do you like to go the aquarium together on Sunday?” | N, haisya-no yoyakusi-ta kara  “Hmm, since (I) have a dentist appointment.” | USi |
|  |  | N, haisya-no yoyakusi-ta-n-da.  “Hmm, (I) have a dentist appointment.” | FSi |
|  |  | N, haisya-no yoyakusi-ta kara -muri.  “Hmm, it’s impossible since (I) have a dentist appointment.” | FSd |
| 10 | Ichigo-no syun rashii-yo. Yūko, kondo-no doyōbi issyoni ichigogari ika-nai?  “It seems strawberries are in season. Yūko, how about going strawberry-picking together this Saturday?” | N, tomodachi-no pathi iku kara.  “Hmm, since (I) am going to a friend's party.” | USi |
|  |  | N, tomodachi-no pathi iku-n-da.  “Hmm, (I) am going to a friend's party.” | FSi |
|  |  | N, tomodachi-no pathi iku kara muri.  “Hmm, it’s impossible since (I) am going to a friend's party.” | FSd |
| 11 | Nami, raigetsu sato senpai-no sotsugyoushiki-da-yo-ne. issyoni mi-ni ika-nai?  “Nami, it's Sato's graduation ceremony next month. Would you like to go seeing it together?” | N, jugyō-no genchi chōsa iku kara.  “Hmm, since (I) am going to do a field survey for a class.” | USi |
|  |  | N, jugyō-no genchi chōsa iku-n-da.  “Hmm, (I) am going to do a field survey for a class.” | FSi |
|  |  | N, jugyō-no genchi chōsa iku kara muri.  “Hmm, it’s impossible since (I) am going to do a field survey for a class.” | FSd |
| 12 | Yūko, kono bijutsomoshiuten omosiro-sō-da-ne. saraisyū hajimat-tara issyoni mi-ni ika-nai?  “Yūko, this art exhibition looks interesting. Would you like to go seeing it together when it starts the week after next?” | N, kyōiku jisshū aru kara.  “Hmm, since (I) have teaching practice.” | USi |
|  |  | N, kyōiku jisshū aru-n-da.  “Hmm, (I) have teaching practice.” | FSi |
|  |  | N, kyōiku jisshū aru kara muri.  “Hmm, it’s impossible since (I) have teaching practice.” | FSd |
| 13 | Nami, shumatsu tanjobi patei suru-nda. Yokat-tara ko-nai?  “Nami, I'm having a birthday party this weekend. Would you like to join us? “ | N, yotei haitteru kara.  “Hmm, since (I) have got plans.” | USi |
|  |  | N, yotei haitteru-n-da.  “Hmm, (I) have got plans.” | FSi |
|  |  | N, yotei haitteru kara muri.  “Hmm, it’s impossible (I) have got plans.” | FSd |
| 14 | Yūko, ima matusimaga kirei rasiiyo. Syūmatu issyoni asobi-ni ika-nai？  “Yūko, I just heard that Matsushima is beautiful now. Would you like to go to visit together this weekend?” | N, kuruma-no kyōsyū aru kara.  “Hmm, since (I) have to go to car school.” | USi |
|  |  | N, kuruma-no kyōsyū aru-n-da.  “Hmm, (I) have to go to car school.” | FSi |
|  |  | N, kuruma-no kyōsyū aru kara muri.  “Hmm, it’s impossible, since (I) have to go to car school.” | FSd |
| 15 | nami, raisyū tesuto owat-tara, gēmusentAa ika-nai？  “Nami, would you like to go to the Game Center after the test next week?” | N, repōto kaka-nakya-ike-nai kara.  “Hmm, since (I) have to write a report.” | USi |
|  |  | N, repōto kaka-nakya-ike-nai-n-da.  “Hmm, (I) have to write a report.” | FSi |
|  |  | N, repōto kaka-nakya-ike-nai kara muri.  “Hmm, it’s impossible, since (I) have to write a report.” | FSd |
| 16 | Kenichi, raisyū-no kinyōbi kanngeikai aru kedo, issyoni sankasi-nai?  “Kenichi, there's a welcome party next Friday, would you like to join us?” | N, syūgatu aru kara.  “Hmm, since (I) have a job searching.” | USi |
|  |  | N, syūgatu aru-n-da.  “Hmm, (I) have a job searching.” | FSi |
|  |  | N, syūgatu aru kara muri.  “Hmm, it’s impossible since (I) have a job searching.” | FSd |
| 17 | Takuya, raigetsu-no kokusai kaigi de boranteia boshūsuru-rasii-yo. Issyoni yattemi-nai?  “Takuya, I heard they're looking for volunteers for the international conference next month. Would you like to try it together?” | N, kenkyūshitsu-no ibento tetsudau kara.  “Hmm, since (I) have to help with a lab event.” | USi |
|  |  | N, kenkyūshitsu-no ibento tetsudau-n-da.  “Hmm, (I) have to help with a lab event.” | FSi |
|  |  | N, kenkyūshitsu-no ibento tetsudau kara muri.  “Hmm, it’s impossible since (I) have to help with a lab event.” | FSd |
| 18 | Kenichi, syashin toru-no jyouzu-da-yone. Issyoni kono kontesuto sankashinai?  “Kenichi, I know you're good at taking pictures. Would you like to participate in this contest together?” | N, bukatsu taihen-da kara.  “Hmm, since (I) am busy with club activities.” | USi |
|  |  | N, bukatsu taihen-nan-da.  “Hmm, (I) am busy with club activities.” | FSi |
|  |  | N, bukatsu taihen-da kara muri.  “Hmm, it’s impossible since (I) am busy with club activities.” | FSd |
| 19 | Takuya, raisyu yakyūdaikai-da-yone. Issyoni oen ika-nai?  “Takuya, there' s a baseball tournament next week, right? Would you like to go cheering together?” | N, tesuto aru kara.  “Hmm, since (I) have a test.” | USi |
|  |  | N, tesuto aru-n-da.  “Hmm, (I) have a test.” | FSi |
|  |  | N, tesuto aru kara muri.  “Hmm, since (I) have a test.” | FSd |
| 20 | Kenichi, manga kenkyukai-ni hairou-to omou-n-da-kedo, issyoni mi-ni ika-nai?  “Kenichi, I’m thinking of joining the manga research group, would you like to go together to take a look?” | N, boutobu-ni hairu kara.  “Hmm, since (I) am going to join the boat club.” | USi |
|  |  | N, boutobu-ni hairu-n-da.  “Hmm, (I) am going to join the boat club.” | FSi |
|  |  | N, boutobu-ni hairu kara muri.  “Hmm, it’s impossible since (I) am going to join the boat club.” | FSd |
| 21 | Raisyū daigakusai aru-ne. Takuya, issyoni mi-ni ikanai?  “There is a university festival next week. Takuya, would you like to go together?” | Aa, onityan-no keikonsiki aru kara.  “Ah, since (I) have to go to my brother's wedding.” | USi |
|  |  | Aa, onityan-no keikonsiki aru-n-da.  “Ah, (I) have to go to my brother's wedding.” | FSi |
|  |  | AAa, onityan-no keikonsiki aru kara muri.  “Ah, it’s impossible since (I) have to go to my brother's wedding.” | FSd |
| 22 | Kyūryou ii baito bosyusiteru-yo. Kenichi, issyoni obosi-nai.  “There' s a part-time job that pays well. Kenichi, would you like to apply with me?” | Aa, kimatus shaken-no junbi aru karu.  “Ah, since (I) have to prepare for the final exam.” | USi |
|  |  | Aa, kimatus shaken-no junbi aru-n-da.  “Ah, (I) have to prepare for the final exam.” | FSi |
|  |  | Aa, kimatus shaken-no junbi aru kara muri.  “Ah, it’s impossible since (I) have to prepare for the final exam.” | FSd |
| 23 | Takuya, syūkatu hajime-nai-to-ike-nai-yone. Kinyōbi-no kigyū setumeikai issyoni ika-nai?  “Takuya, we have to start job hunting, don't we? Would you like to go together to the company information session on Friday?” | Aa, daigakuin-no nyusi setumeikai-ni iku kara.  “Ah, since (I) am going to an admission meeting for graduate school.” | USi |
|  |  | Aa, daigakuin-no nyusi setumeikai-ni iku-n-da.  “Ah, ((I) am going to an admission meeting for graduate school.” | FSi |
|  |  | Aa, daigakuin-no nyusi setumeikai-ni iku kara muri.  “Ah, it’s impossible since (I) am going to an admission meeting for graduate school.” | FSd |
| 24 | Kenichi, kono sisetu kengaku omosiro-sou-da-ne. Raisyu issyoni taikensi-nai?  “Kenichi, this facility tour seems interesting. Would you like to experience it together next week?” | Aa, intansippu aru kara.  “Ah, since (I) have an internship.” | USi |
|  |  | Aa, intansippu aru-n-da.  “Ah, (I) have an internship.” | FSi |
|  |  | Aa, intansippu aru kara muri.  “Ah, it’s impossible since (I) have an internship.” | FSd |
| 25 | Raisyu sakura saju-to omou-yo. Takuya,issyoni hanami-ni iaka-nai?  “I believe the cherry blossoms will bloom next week. Takuya, would you like to go seeing the cherry blossoms together?” | Aa, oya-ga jikka kara kuru kara.  “Ah, since my parents are coming from home.” | USi |
|  |  | Aa, oya-ga jikka kara kuru-n-da.  “Ah, since my parents are coming from home.” | FSi |
|  |  | Aa, oya-ga jikka kara kuru kara muri.  “Ah, it’s impossible since my parents are coming from home.” | FSd |
| 26 | Kenichi, sekkaku-no souritu 100 syūnen-no ensyoukai-da-shi, raisyu issyouni ika-nai?  “Kenichi, since it' s the 100th anniversary concert for the foundation, would you like to go together next Wednesday?” | Aa, sakka-no siai mi-ni iku kara.  “Ah, since (I) am going to watch a soccer game.” | USi |
|  |  | Aa, sakka-no siai mi-ni iku-n-da.  “Ah, (I) am going to watch a soccer game.” | FSi |
|  |  | Aa, sakka-no siai mi-ni iku kara muri.  “Ah, it’s impossible since (I) am going to watch a soccer game.” | FSd |
| 27 | Takuya, raisyu-no kinyōbi, kenkyusitu-no sinenkai aru-yo. Issyoni ika-nai?  “Takuya, there is going to be a new year's party at our lab next Friday. Would you like to go together?” | Aa, sifuto hatteiru kara.  “Ah, since (I) have to take a shift.” | USi |
|  |  | Aa, sifuto hatteiru-n-da.  “Ah, (I) have to take a shift.” | FSi |
|  |  | Aa, sifuto hatteiru kara muri.  “Ah, it’s impossible since (I) have to take a shift.” | FSd |
| 28 | kenichi, naruko-ni kireina kōyō supotto-ga arun-da-kedo, raisyū issyoni ika-nai？  “Kenichi, there's a beautiful spot in Naruko where you can see the autumn leaves, would you like to go together next week?” | Aa, hoka-no hito-to yakusokusityatta kara.  “Ah, since (I) have made an appointment with someone else.” | USi |
|  |  | Aa, hoka-no hito-to yakusokusityatta-n-da  “Ah, (I) have made an appointment with someone else.” | FSi |
|  |  | Aa, hoka-no hito-to yakusokusityatta kara muri.  “Ah, it’s impossible since (I) have made an appointment with someone else.” | FSd |
| 29 | karada ugokas-itai-naa. Takuya, kinyobi basukettoboru yara-nai？  “I really want to exercise. Takuya, would you like to play basketball on Friday?” | Aa, sensei-to mendan aru kara.  “Ah, since (I) have a meeting with my teacher.” | USi |
|  |  | Aa, sensei-to mendan aru-n-da.  “Ah, (I) have a meeting with my teacher.” | FSi |
|  |  | Aa, sensei-to mendan aru kara muri.  “Ah, it’s impossible since (I) have a meeting with my teacher.” | FSd |
| 30 | Aki-ha yappari yama-da-naa. kenichi, issyoni doraibu ika-nai?  “Mountains are the best place to be in autumn. Kenichi, would you like to go for a drive together?” | Aa, syukudai nokotteiru kara.  “Ah, since (I) have homework to finish.” | USi |
|  |  | Aa, syukudai nokotteiru-n-da.  “Ah, (I) have homework to finish.” | FSi |
|  |  | Aa, syukudai nokotteiru kara muri.  “Ah, it’s impossible since (I) have homework to finish.” | FSd |

Table 2. Stimulus dialog sets of request–acceptance/reservation exchange used in the experiment.

Each set consists of two turns of making a request and an acceptance, or a reservation.

| Item | First turn | Second turn | Condition |
| --- | --- | --- | --- |
| 1 | Eigo zenzen jisin-ga nai-n-da. Yūko, eigo-no robun chiekkusite-mora-e-nai?  “I’m not confident in my English at all. Yūko, could you check my English paper for me?” | Aa, mochiron. Yaku-ni tateru nara.  “Ah, sure. If I can be of any help.” | Acceptance |
|  |  | Un, tugo-no ii jikan siraberu-ne.  “Hmm, I have to check on a convenient time.” | Reservation |
| 2 | Pasokon koware-tya-tta-n-da. Nami, issyoukan dake kasite-mora-e-nai?  “My computer broke down. Nami, can I borrow yours for a week?” | Un, ii-yo. Issyoukan-de ii-no?  “Hmm, okay, only one week?” | Acceptance |
|  |  | Aa, tugo kakuninsite utaeru-ne.  “Ah, I will check my availability and let you know.” | Reservation |
| 3 | Rekishi-ni kyoumi-ga aru-n-da. Nami, kondo sono hon kashitte-mora-e-nai?  “I’m interested in history. Nami, could you lend me that book sometime?” | Aa, fun. Ii-yo.  “Ah, hmm. It' s okay.” | Acceptance |
|  |  | Un, tugo-no ii jikan siraberu-ne.  “Hmm, I have to check on a convenient time.” | Reservation |
| 4 | Kopiki-no tukaikata wakan-nai-n-da. Yūko, oshiete-morae-nai?  “I don't know how to use the copier. Yūko, could you show me how?” | Un, ii-yo. Kandan-da-yo.  “Hmm, okay. It's easy.” | Acceptance |
|  |  | Aa, ai-tara oshieru-ne.  “Ah, I will tell you when I’m free.” | Reservation |
| 5 | Nami, raisyu zemi-ni sanka-deki-nai kara, shiryou tot-toite-mora-e-nai?  “Nami, I won't be able to attend the seminar next week, so could you please keep the materials for me?” | Aa, un, wakatta.  “Ah, yeah, I got it.” | Acceptance |
|  |  | Un, sankasuru-kadouka kimattei-nai-yo.  “Hmm, I have not decided whether I will attend or not.” | Reservation |
| 6 | Takuya, nichiyōbi konshinka-nan-dakedo, mise-no yoyakushite-morae-nai?  “Takuya, we have a get-together on Sunday, could you make a reservation for us?” | Un, ii-yo. Makasete.  “Hmm, sure. Let me.” | Acceptance |
|  |  | Aa, jyaa, tore-tara renrakusuru-ne.  “Ah, well, if I get the reservation, I will call you.” | Reservation |
| 7 | Saikin kankokugo-wo benkyoushiteru-n-da. Kenichi, kankokujin-no tomodachi-o syoukaishite-more-nai?  “I’m studying Korean recently. Kenichi, could you introduce me to your Korean friends?” | Un, mochiron. Syoukaisuru-yo.  “Hmm, of course. I will introduce them.” | Acceptance |
|  |  | Aa, ai-tara oshieru-ne.  “Ah, I will tell you when I’m free.” | Reservation |
| 8 | Takuya, raisyu ryouko-no toki, kuruma-ni nosete-morae-nai?  “Takuya, could you give me a ride in your car when we travel next week?” | Un, okke. Issyoni ikou.  “Hmm, okay. Come together.” | Acceptance |
|  |  | Aa, iku-kadouka mada kimara-nakute.  “Ah, I still have not decided whether to go or not.” | Reservation |
| 9 | Takuya, kaze-de mokuyōbi-no jugyō-ni syuseki-deki-nai kara, repoto kawari-ni dashite-morae-nai?  “Takuya, I cannot attend Thursday's class due to a cold, so can you submit my report instead? | Aa, waka-tta. Kaze daijyobu?  “Ah, I got it. you okay with that cold?” | Acceptance |
|  |  | Un, saki-ni sensei-ni kite-ne.  “Hmm, you should talk to the professor first.” | Reservation |
| 10 | Kedai-ga mitukara-nai-n-da. Kenichi, issyoni sagashite-morae-nai?  “I cannot find my phone. Kenichi, could you please look for it together?” | Aa, sorya taihen. Issyoni saga-sou.  “Ah, my God. Let's find it together.” | Acceptance |
|  |  | Un, sakki doko i-tta-no?  “Hmm, where did you go just now?” | Reservation |
| 11 | Kono keki oishii-ne. Yūko, tukurikata oshietekure-nai?  “This cake is delicious. Yūko, could you show me how to make it?” | Aa, un. Yorokode.  “Ah, yeah. Gladly.” | Acceptance |
|  |  | Un, tyotto serishi-tekara oshierune.  “Hmm, I will tell you after I sort out how to make it” | Reservation |
| 12 | Nami, raisyu ryoko-de issyukan rusu-ni suru-n-dakedo, uchi-no inu atukatte-morae-nai?  “Nami, I’m going away on a trip next week for a week, could you take care of my dog?” | Un. Ii-yo. Inu suki-da-shi.  “Hmm. It's okay. I like dogs.” | Acceptance |
|  |  | Aa, oya-ni kite-mite renrakusuru-ne.  “Ah, I will ask my parents and get back to you” | Reservation |
| 13 | Yūko, raigetu-no daigakusai syutuensuru-n-da-kedo, dansu-no shidoshite-morae-nai?  “Yūko, I’m going to perform at the university festival next month, could you instruct me how to dance?” | Un, okke. Ganbatte-ne.  “Hmm, okay. Best of luck to you.” | Acceptance |
|  |  | Aa, sukejuru chiekkushite henjisuru-ne.  “Uh, I'll check my schedule and get back to you.” | Reservation |
| 14 | Nami, piano jyouzu-da-yone. Shinnenkai-de ensoushite-morae-nai?  “Nami, you are good at playing the piano. Could you play for our New Year’s party?” | Un, ii-yo. Watashi-de yokereba.  “Hmm, okay. If it's okay with you.” | Acceptance |
|  |  | Aa, sukejuru mitekara oshieru-ne.  “Ah, I will let you know after I see the schedule.” | Reservation |
| 15 | Yūko, jikken yarou-to omotteru-n-da-kedo, raigetu kyoryokushite-morae-nai?  “Yūko, I’m thinking of doing an experiment, could you help me out next month?” | Aa, syoukai. Apirushi-toku.  “Ah, sure. I will appeal to them.” | Acceptance |
|  |  | Aa, nittei chiekkushite reirakusuru-ne.  “Ah, I will check my schedule and get back to you.” | Reservation |
| 16 | Takuya, konnendo-no buin bosyutyū-nan-da-kedo, uchi-no sakuru kohai-ni apirushite-morae-nai?  “Takuya, we are recruiting members for the club this year, could you appeal our club to juniors?” | Aa, syoukai. Apirushi-toku.  “Ah, sure. I will appeal to them.” | Acceptance |
|  |  | Un, kanjityou-ni kitemi-tekara henjisuru-ne.  “Hmm, let me ask the leader and I’ll get back to you.” | Reservation |
| 17 | Raisyu gakko isogashi-n-da. Kenichi, suyōbi-no shifuto-o kawatte-morae-nai?  “I have lots of school work next week. Kenichi, could you switch shifts together on Wednesday?” | Un, daijyoubu, kawaru-yo.  “Hmm, okay, I could switch.” | Acceptance |
|  |  | Aa, yotei kakuteishite shiraseru-ne.  “Ah, I will check my plans and let you know.” | Reservation |
| 18 | Takuya, raisyū shinseki-ga aso-bi kuru-n-da-kedo, kuruma-o kashite-morae-nai?  “Takuya, my relatives are visiting me next week, could I borrow your car?” | Un, kashite-agetu-to.  “Hmm, I will lend it to you.” | Acceptance |
|  |  | Aa, tugo kakuninshi-tekara tutaeru-ne.  “Ah, I will let you know after I confirm my availability.” | Reservation |
| 19 | Kenichi, saraisyu hikkoshisuru-n-da-kedo, tetutatte-morae-nai?  “Kenichi, I will be moving the week after next, could you help me?” | Un, tatutau-yo.  “Hmm, let me help you.” | Acceptance |
|  |  | Aa, yottei mite henjisuru-ne.  “Oh, I will check my schedule and get back to you.” | Reservation |
| 20 | Takuya, nittyu bunka koryukai-o suru-n-da-kedo, konsyūtyū-ni posuta-no dezainshite-morae-nai?  “Takuya, we are planning to hold a Japan-China cultural exchange meeting, could you design a poster for us by the end of this week?” | Un, ii-yo. Konsyūtyū-da-yone.  “Hmm, fine. It's the end of the week, right?” | Acceptance |
|  |  | Aa, tugo-ga ii-kadouka mitemiru-ne.  “Ah, let me see if that's convenient.” | Reservation |
| 21 | Mensetu sugoku shinpai-nan-da. Yūko, raisyū mogi mensetu tukiatte-morae-nai?  “I’m really worried about the interview. Yūko, could you accompany me to a mock interview next week?” | Un, ii-yo. Konsyūtyū-da-yone.  “Hmm, fine. It's the end of the week, right?” | Acceptance |
|  |  | Aa, tugo-ga ii-kadouka mitemiru-ne.  “Ah, let me see if that's convenient.” | Reservation |
| 22 | Nami, kenkyu-no susumikata moyo-tteru-n-da-kedo, raisyu-no kinyou tyotto Soudan-ni note-morae-nai?  “Nami, I'm not sure how to proceed with my research. Could you give me some advice next Friday?” | Un, taihen-da-ne. issyoni hanasou.  “Hmm, that's tough. Let's talk about it together.” | Acceptance |
|  |  | Aa, nittei mite, konnichityu-ni tutaeru-ne.  “Ah, I will check the schedule and let you know by the end of the day.” | Reservation |
| 23 | Nami, raisyu-no kouryukai-no toki, senpai-no mukae-ni itte-morae nai?  Nami, could you pick up your senior for the social event next week? | Aa, okke. Makasete.  “Ah, okay. I got it.” | Acceptance |
|  |  | Un, sonohi aiteiru-kadouka kakuninsuru-ne.  “Hmm, I will see if I’m free that day.” | Reservation |
| 24 | Yūko, raigetu-no wakusyoppu-no shisyou junbishite-morae-nai?  “Yūko, could you please prepare the materials for next month's workshop?” | Un, daijyoubu-da-yo.  “Yeah, it's okay.” | Acceptance |
|  |  | Aa, kongetu-no nitei mitekara oshieru-ne.  “Uh, I will let you know when I see the schedule for this month.” | Reservation |
| 25 | Nami, syumatu patei yari-tai-nda-kedo, tomodachi sasotte-morae-nai?  “Nami, I want to have a party this weekend, could you invite some friends?” | Aa, mochiron. Patei, daisuki.  “Oh, of course. I love parties.” | Acceptance |
|  |  | Un, dare-ka kangaete-miru-ne.  “Hmm, I'll see who I can find.” | Reservation |
| 26 | Raigaki ryu-kara dasa-nai-to ike-nai-n-da. Takuya, kinyōbi issyoni heya sagashite-morae-nai?  “I’m going to have to move out of the dorm next semester. Takuya, could you look for a room together on Friday?” | Aa, jya, issyoni ikou.  “Uh, well, I will come with you.” | Acceptance |
|  |  | Un, sukejuru kakuninshite tutaeru-ne.  “Hmm, I will check the schedule and let you know.” | Reservation |
| 27 | Kenichi, jikenshitu-no riyou houhou wakara-nai-n-da-kedo, hima-na toki tyotto annaishite-morae-nai?  “Kenichi, I don't know how to use the laboratory, could you guide me when you're free?” | Aa, mochiron. Kinyōbi, dou?  “Ah, of course. How about Friday?” | Acceptance |
|  |  | Un, sukejuru mite-kara oshieru-ne.  “Hmm, I'll let you know when I see the schedule.” | Reservation |
| 28 | Takuya, jidensya koware-tya-tta-n-da-kedo, doyōbi tyotto naoshite-morae-nai?  “Takuya, my bike has broken down, could you fix it for me on Saturday?” | Aa, ii-yo. Doyōbi-no gozentyu-ni shiyou.  “Ah, okay. Let's do it on Saturday morning.” | Acceptance |
|  |  | Un, tugo kakuninshi-tekara renrakusuru-ne.  “Hmm, I'll check my availability and get back to you.” | Reservation |
| 29 | Takuya, raisyu natumesodeki-no dokusyokai-o suru-nda-kedo, shikai-o yatte-morae-nai?  “Takuya, we're having a book club on Soseki Natsume next week, could you be the host?” | Un, daijyoubu-da-to omou-yo.  “Hmm, I think I'll be fine.” | Acceptance |
|  |  | Aa, raisyu-no sukejuru-ga aiteiru-ka miru-ne.  “Ah, I'll see if my schedule is free next week.” | Reservation |
| 30 | Kenichi, raisyu-no suiyōbi-madeni, kondo-no shiai aite-no jyoho seirishite-morae-nai?  “Kenichi, could you please sort out the information about our upcoming opponent by next Wednesday?” | Un, waka-tta. Suiyōbi-made-ne.  “Hmm, okay. Until Wednesday, right?” | Acceptance |
|  |  | Un,nittei shirabete-kara henjisuru-ne.  “Hmm, I'll check the dates and get back to you” | Reservation |
